# Management of infections caused by carbapenemase-producing Enterobacterales in France: a real-world study

**DOI:** 10.1093/jacamr/dlaf260

**Published:** 2026-01-09

**Authors:** B Pilmis, L Escaut, M Seguret, L Weber, F Bussy, S Figueiredo, L Dortet

**Affiliations:** Department of Clinical Microbiology, Saint-Joseph & Marie-Lannelongue Hospitals, Paris, France; Micalis Institute UMR 1319, Université Paris-Saclay, INRAe, AgroParisTech, Orsay, France; Faculty of Medicine, Team ‘Resist’ UMR1184 ‘Immunology of Viral, Auto-Immune, Hematological, and Bacterial Diseases (IMVA-HB),’ INSERM, Université Paris-Saclay, CEA, Le Kremlin-Bicêtre, France; Department of Infectious and Tropical Diseases, Assistance Publique–Hôpitaux de Paris, Bicêtre Hospital, Paris-Saclay University, Le Kremlin-Bicêtre, France; Department of Anesthesia and Surgical Intensive Care, Bicêtre Hospital, Paris-Saclay University, Assistance Publique–Hôpitaux de Paris, Le Kremlin-Bicêtre, France; Department of Anesthesia and Surgical Intensive Care, Bicêtre Hospital, Paris-Saclay University, Assistance Publique–Hôpitaux de Paris, Le Kremlin-Bicêtre, France; Department of Bacteriology-Hygiene, Bicêtre Hospital, Assistance Publique–Hôpitaux de Paris, Faculty of Medicine and Paris-Sud University, le Kremlin-Bicêtre, France; Department of Anesthesia and Surgical Intensive Care, Bicêtre Hospital, Paris-Saclay University, Assistance Publique–Hôpitaux de Paris, Le Kremlin-Bicêtre, France; Faculty of Medicine, Team ‘Resist’ UMR1184 ‘Immunology of Viral, Auto-Immune, Hematological, and Bacterial Diseases (IMVA-HB),’ INSERM, Université Paris-Saclay, CEA, Le Kremlin-Bicêtre, France; Department of Bacteriology-Hygiene, Bicêtre Hospital, Assistance Publique–Hôpitaux de Paris, Faculty of Medicine and Paris-Sud University, le Kremlin-Bicêtre, France; Associated French National Reference Center for Antibiotic Resistance: Carbapenemase-Producing Enterobacterales, Le Kremlin-Bicêtre, France

## Abstract

**Background:**

Carbapenemase-producing Enterobacterales (CPE) are a significant public health concern, with limited treatment options and high mortality rates. The epidemiology and management of CPE infections in France remain insufficiently documented.

**Objectives:**

To provide a multicentre snapshot of the management of CPE infections in France, assess current clinical practices and evaluate their alignment with international guidelines.

**Methods:**

We conducted a multicenter, observational, cross-sectional study across 31 French hospitals between September 2021 and March 2023. Adult patients with confirmed CPE infections were included. Clinical and microbiological data were collected retrospectively. Treatment regimens were analysed, and therapeutic appropriateness was assessed based on European and international guidelines.

**Results:**

Among 6936 screened patients, 96 met the inclusion criteria. The most frequently isolated pathogens were *Klebsiella pneumoniae* (42%), *Escherichia coli* (18%) and *Enterobacter cloacae* complex (16%). OXA-48-like carbapenemases were predominant (50%), followed by New Delhi metallo-β-lactamase (NDM) (38%). Initial empirical therapy was administered in 92.7% of patients overall; appropriateness was 50% in OXA-48-like infections and 25.5% in NDM infections. On multivariable analysis, 30-day mortality (34%) was associated with comorbidity burden and septic shock, whereas neither time to appropriate therapy nor therapy appropriateness independently predicted death. These data suggest outcomes were mainly driven by patient factors and severity, despite low empirical appropriateness—particularly for NDM—which supports efforts to improve early targeting through rapid diagnostics.

**Conclusions:**

This study highlights the challenges associated with CPE infections, including high mortality and frequent inappropriate empirical therapy. Our findings emphasize the need for optimized antimicrobial stewardship and rapid microbiological diagnostics to improve patient outcomes.

## Introduction

Multidrug-resistant organisms, particularly carbapenemase-producing Enterobacterales (CPE), are an increasing public health concern worldwide and represent one of the most concerning issues in the field of antimicrobial resistance.^1^ There are notable geographical disparities in the types of carbapenemase enzymes and resistance mechanisms observed globally. The three main types of carbapenemases are *Klebsiella pneumoniae* carbapenemase (KPC), New Delhi metallo-β-lactamase (NDM) and oxacillinase-48 (OXA-48). KPC enzymes are predominant in North and South America, as well as parts of Southern Europe (especially Italy), where they have been linked to widespread outbreaks in healthcare settings. NDM, first identified in India, remains highly prevalent in the Indian subcontinent and has spread to Europe, Southeast Asia and Africa. OXA-48-like enzymes, known for their silent dissemination due to challenges in detection, are prevalent in North Africa, the Middle East and parts of Europe, including France. These differences in enzyme distribution influence the effectiveness of available therapeutic strategies, complicating infection management and increasing the need for local epidemiological data.^2,3^

Infections caused by these pathogens are associated with significant morbidity and mortality, prolonged hospital stays and elevated healthcare costs. Studies have shown that bloodstream infections (BSIs) caused by CPE have a mortality rate ranging from 30% to 70%, depending on the site of infection, the patient’s comorbidities and the timeliness of effective treatment.^4^ Hospitalized patients with CPE infections experience extended lengths of stay, with durations increasing by an average of 10–15 days compared with infections caused by non-resistant pathogens. This not only contributes to patient morbidity but also imposes a substantial economic burden on healthcare systems, with treatment costs for CPE infections estimated to be two to three times higher than for non-resistant infections.

The Infectious Diseases Society of America (IDSA) and the European Society of Clinical Microbiology and Infectious Diseases (ESCMID) have issued evidence-based guidelines for the management of infections caused by CPE. These guidelines recommend the use of combination therapies and newer agents such as ceftazidime/avibactam, ceftazidime/avibactam plus aztreonam, meropenem/vaborbactam and imipenem/relebactam, cefiderocol, depending on the carbapenemase type. The combination of ceftazidime/avibactam plus aztreonam has been associated with reduced mortality compared with colistin-containing regimens^5^ and is recommended as the first-line option against metallo-beta-lactamase (MBL) producers by currently available guidance and guidelines.^6–8^ International guidelines also emphasize the importance of rapid microbiological testing and antimicrobial stewardship programmes to optimize outcomes.^6–8^

The objectives of this study were to evaluate current clinical practices in the management of CPE infections in France and to assess their alignment with international recommendations. This analysis aims to identify potential gaps and opportunities to improve the treatment of CPE infections in France.

## Material and methods

### Study design

We conducted a multicenter, observational, retrospective study in France between September 2021 and March 2023 (Figure S1). The aim was to describe the management and outcomes of infections caused by CPE in real-life settings.

### Patient identification and microbiological screening

Patients were identified through the French National Reference Center (CNR) for Antibiotic Resistance (Bicêtre Hospital), which centralizes all Enterobacterales isolates suspected of carbapenemase production from both hospital and community laboratories across France. Isolate identification was performed using matrix-assisted laser desorption ionization time-of-flight mass spectrometry (MALDI Biotyper; Bruker Daltonics). Carbapenemase production was confirmed using the NG-Carba5 immunochromatographic assay (NG-Biotech, Guipry, France) and the Carba NP test, as previously described. Short-read next-generation sequencing (NGS) was then performed to determine the exact carbapenemase variant and identify co-produced resistance determinants, including extended-spectrum beta-lactamases (ESBL). Minimum inhibitory concentrations (MICs) were determined by broth microdilution using customized Sensititre plates for last-resort antibiotics (e.g. ceftazidime/avibactam, imipenem/relebactam, meropenem/vaborbactam, aztreonam/avibactam, cefiderocol) as well as for other antibiotics commonly used in the therapeutic arsenal against CPE (e.g. aztreonam, tigecycline, fosfomycin, colistin, meropenem, imipenem, amikacin). Results were interpreted according to the breakpoints established by the European Committee on Antimicrobial Susceptibility Testing (EUCAST).

From this microbiological database, participating hospitals were contacted to retrospectively retrieve the corresponding clinical records.

### Eligibility criteria

Patients were eligible if they were aged ≥18 years and had a documented monomicrobial infection due to a CPE isolate. CPE isolated from screening samples (e.g. rectal swabs) and from urinary or respiratory samples without concomitant bacteraemia were excluded to avoid colonization bias. Accordingly, only urinary and respiratory infections with concurrent bacteraemia were included; other infection sites were included when clinical and microbiological criteria for infection were met. Additional exclusion criteria were: (i) unretrieved medical record at the time of data collection (no access to the patient’s electronic or paper chart, off-site archived record or patient followed exclusively in another institution without available clinical data) and (ii) missing essential information on antibiotic therapy and/or 30-day outcome.

Patients with missing information on antibiotic therapy or with unavailable outcome data were also excluded. Infections were classified according to the Centers for Disease Control and Prevention/National Healthcare Safety Network criteria as BSIs, hospital-acquired pneumonia or ventilator-associated pneumonia (HAP/VAP), urinary tract infections, intra-abdominal infections, skin and soft tissue infections and osteoarticular infections.

### Patient selection flow and participating centres

**Figure 1.**
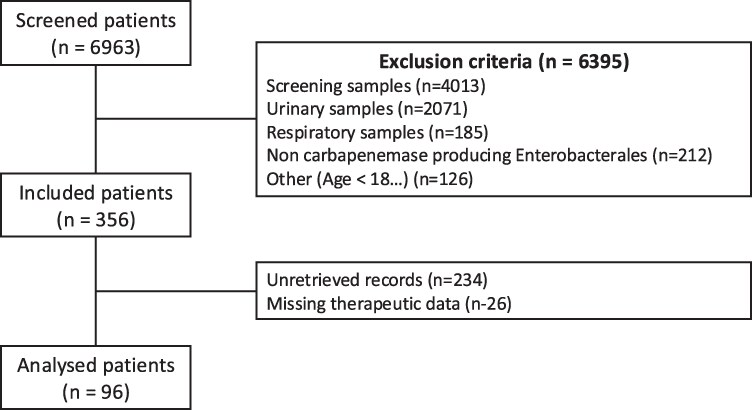
Flow chart.

During the study period, 6963 patients with CPE-positive samples were screened. Among them, 356 fulfilled eligibility criteria. However, 234 medical records could not be retrieved, and 26 lacked essential information regarding antibiotic therapy or outcome, leaving 96 patients for final analysis (Figure 1). Because one of the main objectives of the study was to assess both management practices and 30-day mortality, we applied strict inclusion criteria to avoid incomplete datasets. Cases with missing therapeutic data or missing 30-day outcomes were therefore excluded to ensure robustness and internal consistency of analyses. The majority of excluded cases (234/356, 66%) resulted from inaccessibility of medical records (off-site archives, inter-hospital transfers or missing electronic data) rather than patient- or outcome-related reasons.

Clinical data were collected from 31 hospitals across France, with the number of included patients per centre shown in Figure S1 (available as Supplementary data at *JAC-AMR* Online). To avoid intra-patient clustering, only one index episode per patient was included in the analysis. When multiple infection sites were documented at the index, the episode was classified according to the primary source (with secondary bloodstream infection classified under its source site). Additional CPE episodes occurring after the index event were not analysed as independent observations and were recorded as outcomes (e.g. relapse at day 30). A detailed table of operational case definitions is provided in the Supplementary Methods (Table S2). In France, submission of Enterobacterales isolates suspected of carbapenemase production to the National Reference Center (CNR) is strongly recommended but not mandatory. Consequently, isolated referral practices vary across regions and institutions, resulting in heterogeneous geographical coverage and potential under-representation of certain areas.

### Data collection

For each patient, we collected demographic characteristics, comorbidities, risk factors for CPE acquisition, infection site, severity (including septic shock and ICU admission), empirical and definitive antibiotic regimens (type of molecules, duration, appropriateness according to EUCAST and CASFM recommendations) and outcomes at 30 days. Risk factors for CPE colonization included any of the following: documented prior CPE carriage, hospitalization within the previous 3 months, residence in a long-term care facility, chronic haemodialysis, repeated invasive outpatient procedures or the presence of indwelling medical devices.

### Definitions

Appropriate empirical therapy was defined as the administration of at least one active agent against the infecting isolate within 48 h of infection onset, based on *in vitro* susceptibility testing. Definitive therapy was defined as the targeted regimen administered once microbiological results were available. Ceftazidime/avibactam plus aztreonam was analysed in the subset of MBL-producing infections (NDM or NDM + OXA-48); for OXA-48-like only infections, ceftazidime/avibactam alone was the relevant category.

Definitive antibiotic regimens were classified as colistin-containing regimens, ceftazidime/avibactam or ceftazidime/avibactam plus aztreonam-containing regimens, cefiderocol-containing regimens or other *in vitro* active antibiotics (OAAs). Standard antimicrobial dosages were used, with adjustments for renal function according to manufacturer recommendations. Ceftazidime/avibactam plus aztreonam was administered simultaneously.

Clinical cure was defined as resolution of clinical signs and symptoms of infection without relapse within 30 days after completion of antibiotic therapy.

### Definition of prior CPE colonization

Prior known CPE colonization was defined as any documented carriage of a CPE isolate before the index infectious episode, irrespective of the sampling site (rectal swab, urine, respiratory specimen or wound).

Screening practices were not standardized across participating centres: rectal screening was routinely performed in ICUs, but not systematically in medical or surgical wards, and screening frequency varied between institutions. Therefore, undocumented colonization may have occurred.

### Ethics

The study was approved by the Comité d’Éthique CER-MIT (approval number 2022-0303). Patients were informed by letter (mail or email), and all data were anonymized prior to analysis.

### Statistical analysis

Descriptive statistics were used to summarize the data. Quantitative variables are presented as medians [interquartile range] and categorical variables as numbers (%). Comparisons between groups were performed using Fisher’s exact test for categorical variables and the Mann–Whitney *U*-test for continuous variables.

To identify factors associated with 30-day mortality, univariable analyses were first conducted for relevant clinical and microbiological variables (including comorbidities, site of infection, septic shock, time to effective therapy and appropriateness of empirical and definitive treatment). Variables with *P* < 0.10 were entered into a multivariable logistic regression with backward stepwise selection, retaining variables with *P* < 0.05. Given the limited number of events, multivariable models were restricted to a predefined parsimonious set of clinically relevant predictors (Charlson comorbidity index, septic shock and source of infection), in order to avoid overfitting.

Results are reported as adjusted odds ratios (aORs) with 95% confidence intervals (CIs). Statistical significance was defined as a two-tailed *P* value <0.05. Analyses were performed using R software (version 4.4.2).

## Results

### Study population

Among 6936 screened patients, 356 met the inclusion criteria, and 96 were analysed. The flowchart of the study is presented in Figure 1.

The median age of the patients was 71 years, and 66% were male. Sixty-five (68%) of infections were nosocomial. The main comorbidities identified were cancer, diabetes mellitus and immunodeficiency observed in 33%, 25% and 15% of cases, respectively. The median Charlson comorbidity score was 4.5. Among patients with infections caused by CPE, 74% had at least one risk factor for CPE colonization. A significant proportion of patients were hospitalized in medical wards (46%), while 36% were admitted to the ICU. Twenty-four per cent of patients presented with septic shock, and the 1-month mortality rate was 34%. Two patients (2.1%) experienced a relapse of CPE infection within 30 days. Patient characteristics are detailed in Table 1.

**Table 1.** Patients’ characteristics

Characteristics	Total (*n* = 96)
Male sex, *n* (%)	63 (66)
Age, median (min-max)	71 (22–93)
Comorbidities, *n* (%)	
Cardiovascular	50 (52)
Cancer	32 (33)
Diabetes mellitus	24 (25)
Immunodeficiency	18 (15)
Charlson score, median (min-max)	4.5 (0–12)
Risk factor for CPE^a^, *n* (%)	71 (74)
Hospitalization department, *n* (%)	
Medical ward	44 (46)
Surgical ward	17 (18)
Intensive care unit	35 (36)
Source of infection, *n* (%)	
Urinary tract infection	23 (24)
Intra-abdominal infection	19 (19.7)
Bone and joint infection	15 (15.5)
Skin and soft tissue infection	15 (15.5)
Respiratory tract infection	10 (10)
Catheter-related infection	8 (8.3)
Primary bacteraemia	7 (7)
Septic shock, *n* (%)	23 (24)
Source control procedure, *n* (%)	33 (34)
Relapse within 30 days, *n* (%)	2 (2.1)
One-month mortality, *n* (%)	33 (34)

Risk factors for CPE colonization included previous CPE carriage, recent hospitalization (≤3 months), long-term care facility residence, chronic haemodialysis, repeated invasive procedures or presence of indwelling medical devices.

^a^Carbapenemase-producing Enterobacterales.

Among the 31 community-onset infections, 12 (38.7%) occurred in residents of long-term care facilities. The remaining community-onset cases all involved patients with recent healthcare contacts, including hospitalization within the previous 3 months, chronic haemodialysis or repeated outpatient medical procedures. No case fulfilled the criteria for a strictly community-acquired CPE infection.

### Microbiological data

CPE were primarily cultured from blood cultures (65%), peritoneal samples (13%) and osteoarticular samples (8%). The main source of infection was urinary tract infection-related bacteraemia (24%), intra-abdominal infections (19.7%) and osteoarticular infections (15.5%).

**Figure 2.**
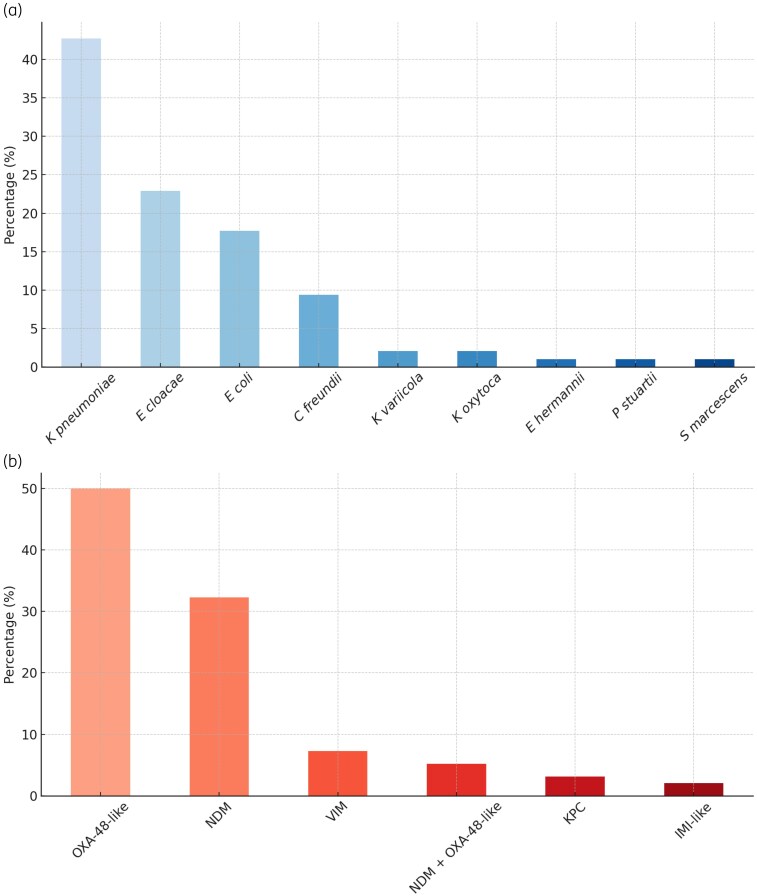
Distribution of CPE according to (a) bacterial species and (b) identified enzyme type.

The predominant carbapenemases identified were OXA-48-like (50%), NDM (38%), VIM (7%), KPC (3%) and IMI (2%). Two isolates (2.1%) co-produced two different carbapenemases. The most frequently isolated bacterial species included *K. pneumoniae* (42%), *E. coli* (18%) and *E. cloacae* complex (16%) (Figure 2). The distribution of specific carbapenemase gene variants identified by NGS is detailed in Figure S2. Susceptibility patterns for tested antibiotics are detailed in Table 2.

**Table 2.** *In vitro* susceptibility of CPE according to EUCAST breakpoints

Antimicrobial agent tested	MIC range (mg/L)	Resistance rate according to EUCAST (%)		
		Global	OXA-48-like	NDM
Aztreonam	≤1 to >32	72.3	65.9	75
Piperacillin/tazobactam	<4 to >32	97.8	100	100
Cefepime	≤1 to >16	76.8	59.6	100
Cefiderocol	≤0.03 to 16	12.3	2.3	22.2
Meropenem	<0.12 to >16	52.6	13.3	100
Meropenem/vaborbactam	≤0.06 to >16	30.1	6.7	100
Eravacycline	0.06 to >8	17.2	15.5	22.2
Tigecycline	>0.5 to >16	32.1	31.1	33.3
Amikacin	≤2 to >32	25.2	19.1	36.1
Colistin	≤0.5 to >16	6.3	4.3	8.3
Cefazidime/avibactam	≤0.5 to >16	45.6	0	100
Ceftazidime/avibactam + aztreonam	≤0.06 to >16	2.1	0	2.7

Regarding antibiotic susceptibility testing (AST), all isolates were resistant to piperacillin/tazobactam. Among NDM-producing isolates, cefiderocol resistance reached 22.2% (Table 2), corresponding to eight resistant strains, predominantly *E. coli*. This is consistent with national data showing lower cefiderocol susceptibility in *E. coli* (≈45%) than in non-*E. coli* Enterobacterales (≈77%), largely driven by the emergence of lineages carrying NDM-5, PBP3 insertions and CMY-type cephalosporinases.

We also identified one *E. coli* isolate resistant to the aztreonam/avibactam combination. Whole-genome sequencing revealed that this strain combined three resistance mechanisms known to reduce susceptibility to both cefiderocol and aztreonam/avibactam: production of NDM-5, a PBP3 insertion and co-production of a CMY β-lactamase. Such multidimensional resistance profiles remain rare but are increasingly reported in high-risk *E. coli* clones.

Overall, susceptibility to aztreonam/avibactam remained high among tested isolates (≈95%), and dual non-susceptibility to cefiderocol and aztreonam/avibactam was exceptional. Susceptibility to colistin also remained high (91.7%).

OXA-48-like-producing isolates were universally susceptible to ceftazidime/avibactam in our cohort (100%). Resistance rates to cefiderocol, colistin and amikacin were observed in 2.3%, 4.3% and 19.1% of tested strains.

### Treatment regimens

Empirical therapy was administered in 92.7% of cases. Appropriateness was higher for OXA-48-like infections than for NDM infections (50.0% versus 25.5%; *P* = 0.019; OR 2.87, 95% CI 1.1–7.9). Appropriateness also varied by site of care, being higher in ICU patients than in those managed on medical/surgical wards (51.4% versus 31.1%). Prior known CPE colonization was not associated with a higher rate of appropriate empirical therapy (45.5% versus 39.5%; *P* = 0.96). By site of infection, empirical appropriateness was 46.2% for digestive infections, 43.5% for urinary infections, 33.3% for osteoarticular infections, 20% for skin/device-related infections and 50% for pulmonary infections (global *P* = 0.41).

**Figure 3.**
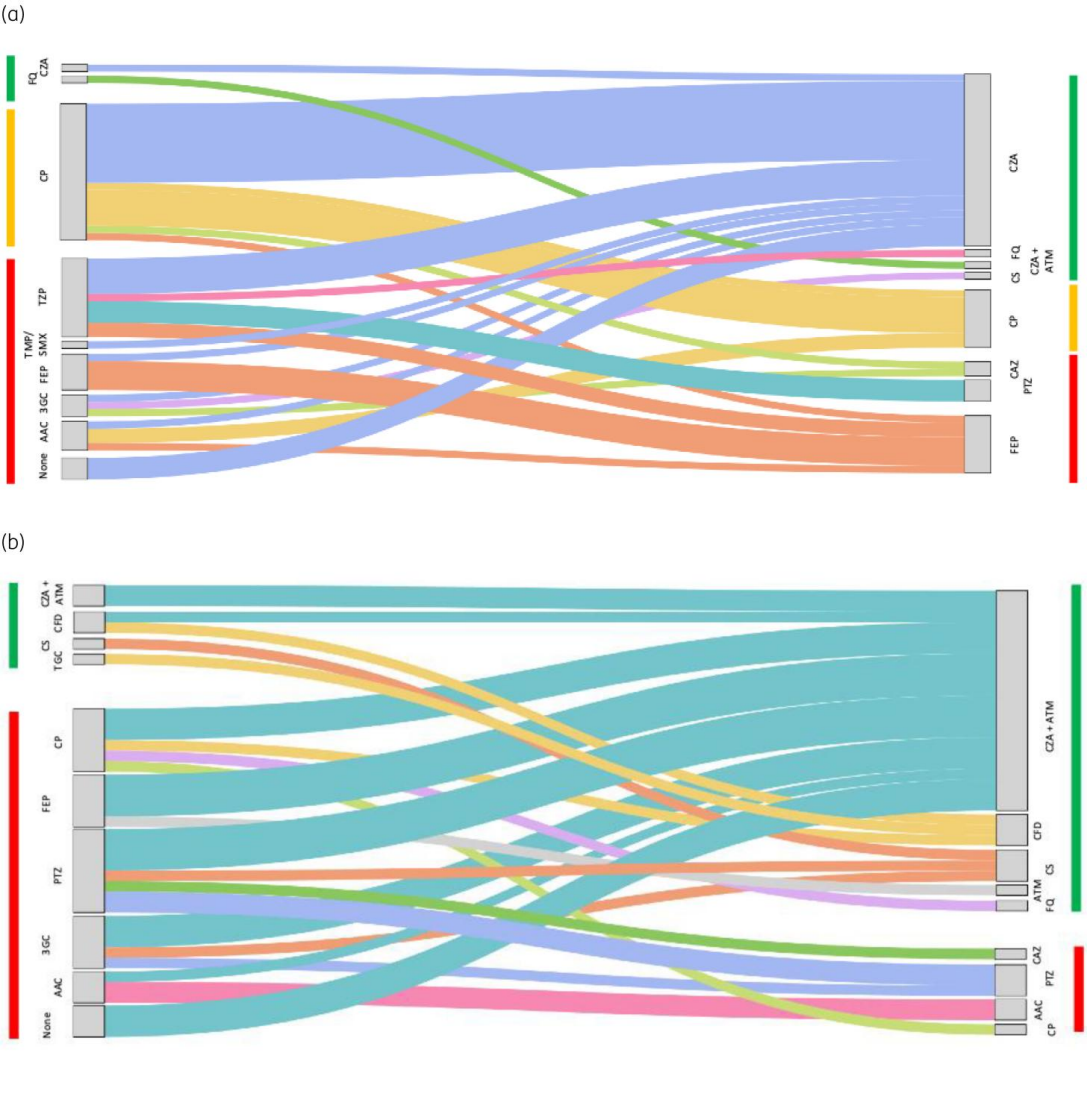
Transition from empirical to definitive therapy is prescribed for infections caused by CPE. (a) OXA-48-like producers (*n* = 48). (b) Metallo-β-lactamase (NDM ± OXA-48) producers (*n* = 36). Each flow represents the number of patients transitioning from a given empirical regimen to a given definitive regimen, with the width of the streams proportional to the absolute number of patients. The green vertical bars represent effective treatments. The orange vertical bars represent treatments that are sometimes effective. The red vertical bars represent ineffective treatments. AAC, amoxicillin/clavulanate; ATM, aztreonam; CFD, cefiderocol; CAZ, ceftazidime; CZA, ceftazidime/avibactam; CP, carbapenem; CS, Colistin; FQ, Fluoroquinolones; PTZ, piperacillin/tazobactam; TGC, tigecycline; 3GC, third generation cephalosporin (cefotaxime or ceftriaxone); FEP, cefepime.

Definitive therapy predominantly included ceftazidime/avibactam for OXA-48-like infections, while MBL-producing infections (NDM or NDM + OXA-48) were treated with ceftazidime/avibactam plus aztreonam (Table 3). Therapeutic adequacy was achieved in 91.7% of OXA-48 cases and 88.3% of NDM cases. The average time to appropriate therapy was 3.75 days for OXA-48 and 4.5 days for MBL infections (*P* = 0.19). Surgical intervention was required in 34.3% of cases. These procedures represented formal source control interventions for intra-abdominal, osteoarticular or device-related infections. The transition from empirical to definitive therapy was visualized using a Sankey diagram (Figure 3). Among the nine patients who did not receive definitive therapy, three had catheter-related BSIs managed by catheter removal alone; four had non-bacteraemic intra-abdominal infections managed surgically (intraoperative sampling results not considered) and the remaining two had limitations in the intensity of care (palliative/comfort-focused management).

**Table 3.** Targeted antibiotic regimens according to carbapenemase type

Targeted regimen	OXA-48-like (*n* = 48)	NDM ± OXA-48 (*n* = 36)
Ceftazidime/avibactam monotherapy, *n* (%)	32 (66.7%)	0 (0%)
Ceftazidime/avibactam + aztreonam, *n* (%)	0 (0%)	28 (77.8%)
Cefiderocol, *n* (%)	6 (12.5%)	4 (11.1%)
Colistin-containing regimens, *n* (%)	3 (6.3%)	2 (5.5%)
Other active agents (tigecycline, aminoglycosides, fosfomycin), *n* (%)	5 (10.4%)	2 (5.5%)
No targeted therapy (surgery or catheter removal only), *n* (%)	2 (4.1%)	0 (0)

### Outcome

The overall 30-day mortality was 34%. When examined in more detail, mortality was 41.7% for OXA-48-like producers (20/48), 30.6% for NDM producers (11/36), 42.9% for VIM producers (3/7) and 0% for both KPC (0/3) and IMI producers (0/2). The two isolates co-producing two carbapenemases were too few to be analysed as a separate category and were classified according to the dominant enzyme identified. As expected, given the small subgroup sizes, none of these differences reached statistical significance in univariable analysis (all *P* values > 0.1).

When examining the impact of time to appropriate therapy, early administration within <24 h was associated with a significantly lower 30-day mortality (21.1%, 8/38) compared with treatment initiated ≥24 h (**43.1%**, 25/58) (OR 0.35, 95% CI 0.13–0.91; *P* = 0.03).

Using a 48-h threshold, mortality was 38.6% (22/57) among patients receiving appropriate therapy within <48 h and 28.2% (11/39) among those treated ≥48 h (OR 1.60, 95% CI 0.67–3.79; *P* = 0.25).

Thirty-day mortality according to targeted therapy was 25.0% (8/32) in patients receiving ceftazidime/avibactam monotherapy, 28.6% (8/28) in those treated with ceftazidime/avibactam plus aztreonam, 37.5% (3/8) in patients receiving cefiderocol, and 40.0% (2/5) among those treated with colistin-containing regimens. These differences were not statistically significant, reflecting the small number of patients in several therapeutic subgroups. The highest mortality rates were observed in patients presenting with septic shock (47% versus 11%; *P* < 0.001) and in those with intra-abdominal infections (63.1% versus 28.5%; *P* = 0.007).

In the primary multivariable model including Charlson comorbidity index, septic shock and site of infection (urinary tract as the reference), both the Charlson index (adjusted OR 1.52, 95% CI 1.23–1.87; *P* = 0.0001) and septic shock (adjusted OR 3.96, 95% CI 1.24–12.61; *P* = 0.021) were independently associated with 30-day mortality. Compared with urinary tract infections, digestive infections showed a significant trend towards higher mortality (adjusted OR 5.54, 95% CI 1.17–26.18; *P* = 0.031).

Sensitivity analyses further adjusting for appropriateness of empirical or definitive therapy yielded consistent results: septic shock remained significantly associated with mortality (adjusted OR 3.95 and 3.63, respectively), while neither appropriate empirical therapy (adjusted OR 1.02, 95% CI 0.34–3.09; *P* = 0.97) nor appropriate definitive therapy (adjusted OR 0.65, 95% CI 0.12–3.54; *P* = 0.62) had an independent effect on outcome (Table 4).

**Table 4.** Univariate analysis of risk factors for 30-day mortality

Characteristic	Dead (*n* = 34)	Alive (*n* = 62)	*P* value (univariate analysis)	*P* value (multivariate analysis)	aOR [CI 05%]
Age (years), median [IQR]	70.5 [33–93]	71 [22–88]	0.73		
Male sex, *n* (%)	23 (68)	40 (65)	0.76		
Charlson comorbidity score, median [IQR]	5 [2–12]	4 [0–10]	0.026	0.0001	1.52 [1.23–1.87]
Healthcare-associated infection, *n* (%)	21 (62)	44 (71)	0.36		
Septic shock	16 (47)	7 (11)	< 0.001	0.020	3.96 [1.24–12.6]
Source of infection, *n* (%)					5.55 [1.17–26.22]
Urinary tract infection	4 (12)	19 (31)	0.046	0.151	
Intra-abdominal infection	12 (35)	7 (11)	0.024	0.031	
Bone and joint infection	4 (12)	11 (18)	0.56		
Skin and soft tissue infection	5 (15)	10 (16)	0.89		
Respiratory tract infection	5 (15)	5 (8)	0.32		
Catheter-related infection	1 (3)	7 (11)	0.25		
Primary bacteraemia	4 (12)	1 (2)	0.051		
Type of carbapenemase, *n* (%)^a^					
OXA-48-like	20 (59)	28 (45)	0.28		
VIM	3 (9)	4 (6.5)	0.69		
NDM	11 (32)	25 (40.5)	0.51		
KPC	0 (0)	3 (5)	0.55		
IMI	0 (0)	2 (3)	0.53		
Antibiotic therapy, *n* (%)					
Empirically appropriate antibiotic therapy	13 (38.2)	25 (40.3)	0.84		
Definitive appropriate antibiotic therapy	28 (82.4)	58 (93.5)	0.083		

^a^Two isolates produced two different types of enzymes.

## Discussion

CPE are a major public health concern recognized by the WHO.^9,10^ Our multicenter French study describes the microbiological and clinical features of CPE infections and situates them within a rapidly evolving global epidemiology. In France, CPE infections remain relatively infrequent (≈0.031 per 1000 hospital-days), with *K. pneumoniae* most common, followed by *E. coli*, and an increasing contribution of the *En. cloacae* complex. Internationally, the carbapenemase landscape is shifting: OXA-48-like enzymes predominate around the Mediterranean and parts of Europe, KPC remains dominant in the Americas and NDM producers are expanding across Europe and beyond. This expansion is fuelled by patient mobility, healthcare transfer and the spread of successful high-risk lineages and plasmids; co-carriage of OXA-48-like and NDM is increasingly reported. In our cohort, OXA-48-like remained the most frequent enzyme, yet a notable fraction of infections involved NDM-producing Enterobacterales, underscoring this continental trend. Although clonal expansion of successful lineages (e.g. *K. pneumoniae* ST147, *E. coli* ST167/410) is likely to play a role, our data also support the hypothesis of plasmid-mediated dissemination, consistent with the frequent co-carriage of OXA-48-like and NDM enzymes within different species.^11^ Most infections were healthcare-associated, consistent with known risk factors such as recent hospitalization or residence in long-term care.^12^

Therapeutically, ceftazidime/avibactam retained activity against OXA-48-like producers, whereas management of metallo-β-lactamase (MBL) infections relied on ceftazidime/avibactam plus aztreonam or cefiderocol. Of concern, we observed higher cefiderocol resistance among NDM producers in France, a pattern plausibly linked to the expansion of specific *E. coli* lineages (e.g. ST410, ST167, ST361, ST405) harbouring PBP3 insertions and CMY enzymes on an NDM-5 background, rather than to *K. pneumoniae* ST147 alone.^13,14^ In light of these data and recent literature, we recommend routine MIC-based cefiderocol susceptibility testing (iron-depleted broth microdilution) before use in suspected or confirmed MBL infections, and favour aztreonam-avibactam as empirical therapy in known MBL carriers with severe sepsis/septic shock, reserving cefiderocol for targeted therapy once activity is confirmed. When comparing these real-world therapeutic practices with international guidelines (ESCMID, IDSA), several points of alignment and divergence emerged.^7,8^ As recommended by these societies, ceftazidime/avibactam was the preferred targeted therapy for OXA-48-like producers, and patients with NDM-producing Enterobacterales predominantly received ceftazidime/avibactam plus aztreonam. However, empirical therapy frequently lacked activity, especially in NDM infections, reflecting a gap between guideline-based principles and real-world practice. This discrepancy likely stems from limited early identification of patients at risk for CPE infection, inconsistent integration of colonization data outside ICUs and delayed availability of advanced susceptibility testing. These findings highlight the need for improved early recognition and expanded access to rapid diagnostic methods to better align clinical practice with current recommendations.

Clinical outcomes were poor, with a 30-day mortality of 34%, consistent with prior studies.^4^ Mortality was not linked to carbapenemase type but was independently associated with comorbidity burden (Charlson index) and septic shock in multivariable analysis and was higher for non-urinary sources (notably intra-abdominal and other non-urinary infections), which often involve higher inocula and frequently require procedural source control.^4,15^ Importantly, only about half of patients received appropriate empirical therapy, with marked heterogeneity across care settings: appropriateness was higher in the ICU than in medical/surgical wards (51.4% versus 31.1%). In France, rectal screening for CPE is usually performed in ICUs, and colonization status is not systematically integrated into empirical choices outside the ICU; these practice gaps likely contributed to delays in effective therapy and may have adversely impacted outcomes, as suggested elsewhere.

Laboratory implementation also remains a bottleneck. Turnaround times for advanced susceptibility testing of last-resort agents (e.g. aztreonam-avibactam, cefiderocol) and the need for reference methods can delay optimization of therapy. The wider dissemination of standardized protocols and systematic susceptibility testing at the time of CPE identification is essential to improve initial management.

This study has several limitations. First, its retrospective design entails a risk of missing data. A large proportion of otherwise eligible cases (234/356, 66%) could not be included because the corresponding clinical records were unavailable (off-site archiving, inter-hospital transfers or incomplete electronic files). Although these exclusions were mainly due to structural constraints rather than patient-related factors, they inevitably reduce representativeness and may limit the generalizability of our findings at the national level.

Second, screening practices for CPE colonization were not standardized across centres. Systematic rectal screening was largely restricted to ICUs, leading to potential under-detection of colonization in other wards and to possible misclassification bias when evaluating the relationship between colonization status and empirical therapy.

Third, some carbapenemase subgroups (e.g. VIM, KPC and IMI producers) included only a small number of cases, which limits the statistical power to detect outcome differences between enzyme types.

Fourth, because follow-up was limited to 30 days by design, late infectious episodes (>30 days) could not be evaluated. Only two relapses were identified during the 30-day follow-up period, and these were captured as outcomes rather than as new independent episodes.

Finally, the submission of suspected CPE isolates to the French National Reference Center—although strongly encouraged—is not mandatory. Combined with the absence of population denominators for participating hospitals, this heterogeneity in geographical coverage prevented reliable incidence estimation from our dataset.

Despite these limitations, the centralized microbiological confirmation by the National Reference Center and the multicenter clinical data collection provide a robust and valuable real-world snapshot of the current management of CPE infections in France.

In summary, outcomes of CPE infections in France appear driven primarily by host factors and acute severity against a backdrop of shifting carbapenemase epidemiology marked by the rise of NDM producers and lineage-specific resistance phenotypes. Improving early risk recognition, systematic screening and standardized susceptibility testing—particularly for cefiderocol and aztreonam-avibactam—will be essential to optimize empirical choices and patient outcomes as the global landscape continues to evolve.

## Conclusion

In summary, this study provides valuable clinical characteristics, management and treatment outcomes of CPE-related infections. Our findings underline the importance of infection control strategies, particularly in identifying high-risk patients to avoid inadequate empirical antibiotic therapy in this population. The study highlights the high mortality rate associated with CPE-related infections, emphasizing the importance of early recognition and management of infections outside the urinary tract. Furthermore, this high case fatality rate underlines the need to improve prompt diagnosis and treatment strategies.

## Supplementary Material

dlaf260_Supplementary_Data

## Supplementary data

Figures S1 and S2 and Tables S1 and S2 are available as Supplementary data at *JAC-AMR* Online.
