# Supplementary material for: Management of infections caused by carbapenemase-producing Enterobacterales in France: a real-world study
Source: JAC Antimicrob Resist. 2026 Jan 9;8(1):dlaf260. doi: 10.1093/jacamr/dlaf260 (PMC12784193; doi:10.1093/jacamr/dlaf260)
Supplement: dlaf260_Supplementary_Data [file dlaf260_supplementary_data.docx]

**SUPPLEMENTARY DATA**

**Figure S1.** Geographical Distribution of Analyzed Specimen Origins (Each circle represents a specific location from which samples were collected, with the size of the circle corresponding to the number of specimens obtained from that area).

**Figure S2.** Heatmap showing the distribution of carbapenemase gene variants detected by next-generation sequencing (NGS) among Enterobacterales clinical isolates. Species are ordered from the most to the least frequently isolated. Numbers within cells indicate the count of isolates carrying each variant.

**
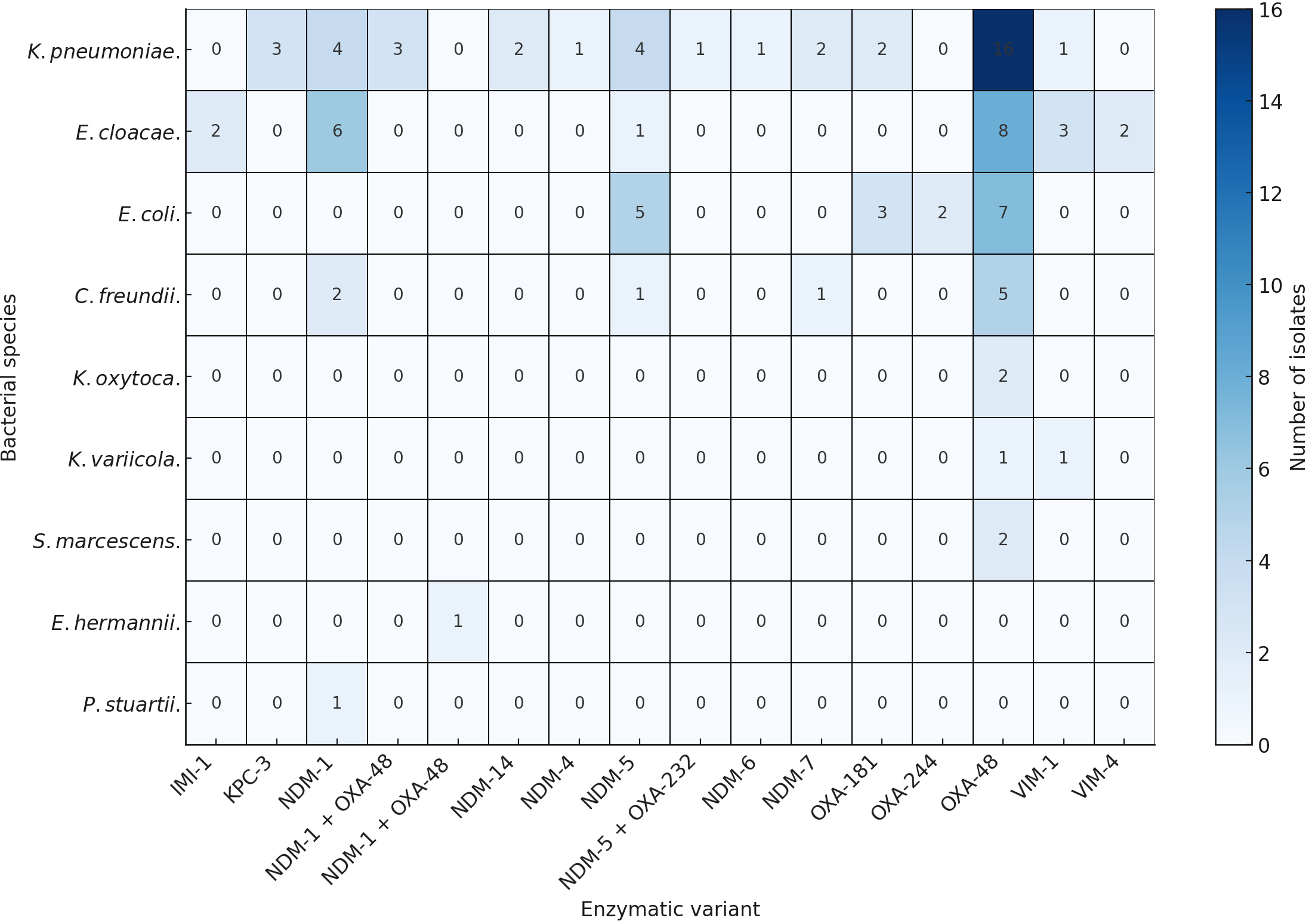
**

**Table S1. List of participating centers (n=31)**

| **Participating centers** |
| --- |
| CHU Angers |
| Hôpital Avicenne |
| CHU Clermont Ferrand |
| CHU Henri Mondor |
| CHU Kremlin Bicêtre  CHU Lille |
| CHU Nancy |
| CHU Orléans |
| CHU Purpan |
| CHU Rennes |
| CHU Saint Denis de la Réunion |
| CHU Strasbourg  CHU Tours |
| Hôpital Cochin |
| Hôpital Saint Antoine  CH Albertville |
| CH Alpes Leman |
| CH Argenteuil |
| CH Bastia |
| CH Boulogne sur Mer  CH Bourgoin  CH Cambrai  CH Flers  CH La Roche sur Yon  CH Libourne  CH Metz  CH Orsay  CH Valenciennes  CH Vannes |
| Hôpital d’instruction des armées Percy |
| Hôpital Foch |

**Table S2.** Operational case definitions and episode

| **Infection type** | **Primary reference** | **Operational definition used in this study** | **Microbiological requirement** | **Exclusions / notes** | **Mapping to analysis groups** |
| --- | --- | --- | --- | --- | --- |
| Bloodstream infection (primary) | CDC/NHSN | Compatible clinical syndrome and at least one positive blood culture for CPE, with no qualifying source at another site. | ≥1 blood culture positive for CPE. | If another site met criteria, BSI was classified as secondary and attributed to that source. | BSI |
| Bloodstream infection (secondary) | CDC/NHSN | Blood culture positive for CPE with a concurrent qualifying infection at a defined source site. | Blood CPE and source site meeting its definition. | Counted under the source (not as primary BSI). | Counted with source (e.g., URINARY, DIGESTIVE, OTHER) |
| Urinary tract infection (catheter or non-catheter) | CDC/NHSN (SUTI) | Compatible urinary symptoms or sepsis of urinary origin and qualifying urine culture; bacteremia, if present, considered secondary to UTI. | Urine culture with CPE from a clinically obtained sample. | Asymptomatic bacteriuria and screening-only samples excluded. | URINARY |
| Hospital-acquired / ventilator-associated pneumonia | CDC/NHSN (HAP/VAP) | New/progressive radiographic infiltrate plus clinical criteria (e.g., fever/leukocytosis/purulent secretions) with microbiological support from lower respiratory tract or blood. | BAL/ETA or blood with CPE. | Upper airway colonization without clinical/radiologic criteria excluded. | OTHER (respiratory) |
| Intra-abdominal infection (including biliary) | Harmonized clinical definition | Compatible abdominal symptoms/signs and imaging or intra-operative evidence of infection; source control recommended when feasible. | Culture from intra-abdominal site/bile or blood with abdominal focus. | Colonization from drains without clinical/imaging evidence excluded. | DIGESTIVE |
| Osteoarticular infection (native or prosthetic) | Harmonized clinical definition (IDSA-compatible) | Clinical and/or imaging features plus microbiology from bone, synovial fluid, deep tissue, or blood with osteoarticular focus. | Culture from bone/synovial/deep tissue or concordant blood. | Superficial swabs excluded. | OTHER (osteoarticular) |
| Skin and soft-tissue infection (incl. device/mesh) | Harmonized clinical definition (IDSA-compatible) | Clinical signs with microbiology from deep tissue/aspirate, or blood with SSTI focus. | Culture from deep specimen or concordant blood. | Superficial swabs alone excluded. | OTHER (skin/soft tissue) |
| Catheter-related infection (CRBSI/CLABSI) | CDC/NHSN / IDSA | Local signs and/or bloodstream infection with catheter involvement (e.g., differential time to positivity or catheter tip culture). | Blood CPE with catheter evidence; if insufficient proof, counted as primary BSI. | If clear non-catheter source existed, attributed to that source. | OTHER (device-related) |
